# Fecal Microbiota Transplantation: Insights into Colon Carcinogenesis and Immune Regulation

**DOI:** 10.3390/jcm13216578

**Published:** 2024-11-01

**Authors:** Olga Brusnic, Danusia Onisor, Adrian Boicean, Adrian Hasegan, Cristian Ichim, Andreea Guzun, Radu Chicea, Samuel Bogdan Todor, Bogdan Ioan Vintila, Paula Anderco, Corina Porr, Horatiu Dura, Sorin Radu Fleaca, Adrian Nicolae Cristian

**Affiliations:** 1 Department of Gastroenterology, University of Medicine, Pharmacy, Science, and Technology of Targu Mures, 540142 Târgu Mures, Romania; brusnic_olga@yahoo.com (O.B.); halalisan5@yahoo.com (D.O.); 2 Faculty of Medicine, Lucian Blaga University of Sibiu, 550169 Sibiu, Romania; adrian.boicean@ulbsibiu.ro (A.B.); adrian.hasegan@ulbsibiu.ro (A.H.); andreea1239@yahoo.com (A.G.); radu.chicea@ulbsibiu.ro (R.C.); bogdan.vintila@ulbsibiu.ro (B.I.V.); paula.anderco@ulbsibiu.ro (P.A.); corina_sibiu@yahoo.com (C.P.); horatiu.dura@ulbsibiu.ro (H.D.); radu.fleaca@ulbsibiu.ro (S.R.F.); adrian.cristian@ulbsibiu.ro (A.N.C.)

**Keywords:** colorectal cancer, fecal microbiota transplantation, microbiome

## Abstract

Colorectal cancer (CRC) constitutes a significant global health challenge, with recent studies underscoring the pivotal role of the gut microbiome in its pathogenesis and progression. Fecal microbiota transplantation (FMT) has emerged as a compelling therapeutic approach, offering the potential to modulate microbial composition and optimize treatment outcomes. Research suggests that specific bacterial strains are closely linked to CRC, influencing both its clinical management and therapeutic interventions. Moreover, the gut microbiome’s impact on immunotherapy responsiveness heralds new avenues for personalized medicine. Despite the promise of FMT, safety concerns, particularly in immunocompromised individuals, remain a critical issue. Clinical outcomes vary widely, influenced by genetic predispositions and the specific transplantation methodologies employed. Additionally, rigorous donor selection and screening protocols are paramount to minimize risks and maximize therapeutic efficacy. The current body of literature advocates for the establishment of standardized protocols and further clinical trials to substantiate FMT’s role in CRC management. As our understanding of the microbiome deepens, FMT is poised to become a cornerstone in CRC treatment, underscoring the imperative for continued research and clinical validation.

## 1. Introduction

Colorectal cancer, a malignancy of the digestive system, has a significant impact on global life expectancy. It ranks among the top three most prevalent cancers with 1.9 million cases annually and 935,000 deaths [1]. In 2020, prostate cancer was the most prevalent form of cancer among men in some Eastern European countries, followed by lung and colorectal cancer. Among women, colorectal cancer ranked second, following breast cancer [2,3,4].

Colorectal cancer exhibits a high resistance to conventional therapies, which complicates and limits treatment options. This underscores the need for targeted, personalized treatment approaches, particularly due to its aggressive nature and high recurrence rate [5,6]. Recent studies have increasingly concentrated on the microbiome and its connection to population health, with a particular focus on its role in various digestive cancers [7]. Bidirectional connections between bacteria and their host are maintained by abundant extracellular vesicles (Gm-EVs) produced in the intestinal microbiota [8]. Proinflammatory bacteria, viruses, fungi and bacteriophages play a key role in mediating inflammatory responses in gastrointestinal pathologies, such as colorectal cancer, inflammatory bowel disease and pancreatic ductal adenocarcinoma. The microbiome composition, influenced by dietary intake of fats and fiber, physical activity and mental health, plays a crucial role in modulating antitumor activity and the risk of cancer development [9,10,11]. Furthermore, the microbiota produces water-soluble B vitamins, vitamin K, endocrine factors and neurotransmitters that influence the central nervous system, affecting cognition and behavior [12]. To restore the physiological intestinal flora and enhance microbiome diversity, fecal microbiota transplantation (FMT) has emerged as a promising solution. This has been implemented in a modern form since the second half of the 20th century and has gained significant interest in recent years [13].

The key conditions for any transplant include strict selection of a compatible microbiome, a healthy donor, an appropriate recipient and the exclusion of pathogens such as bacteria, viruses and infections that could harm the susceptible patient. Additionally, maintaining the host’s homeostasis is crucial [14].

Immune response regulation involves several mechanisms, among which key factors include competition for nutrients, the limitation of pathogen growth and the prevention of pathogen spread through the immune response generated by the association between a healthy microbiome and the host [15].

So far, the most common application of fecal microbiota transplantation (FMT) is in the treatment of *Clostridioides difficile* infections, which frequently arise as a consequence of antibiotic use [16,17]. However, its use extends beyond this application, showing potential benefits in managing various other conditions, including colorectal cancer, ulcerative colitis, Crohn’s disease, metabolic syndrome, obesity and autism [18]. Compared to other organ transplants, FMT offers a unique safety advantage. While it functions similarly to an organ transplant, it cannot be rejected by the body and is seamlessly integrated into the existing microbiota.

After selecting the donors, an essential step involves the preparation of the biomaterial before transplantation. The primary methods of administering FMT include upper digestive routes, such as oral capsules, nasogastric tubes, nasoduodenal or nasojejunal delivery and lower digestive routes, which include colonoscopy, enemas and colonic transendoscopic enteral tubes (TETs). The TET method offers a distinct advantage by allowing repeated transplants over a specified period and facilitating the distribution of the transplant material throughout the entire colon, from the anus to the cecum [19].

The diversity of the intestinal flora influences both the progression and the regression of colorectal cancer. Hence, the bacteria that can be found in the microbiome, the *Enterobacteriaceae family*, *Escherichia coli* and *Enterotoxigenic Bacteroides fragilis*, have a proinflammatory role and promote tumor proliferation, while *Lactobacilii*, *Faecalibaculum rodentium*, *Bifidobacteria* and the *Ruminococcaceae family* are relevant in antitumoral defense and lowering proinflammatory cytokines, controlling cellular DNA degradation. Tumor growth is significantly influenced by short-chain fatty acids produced by beneficial bacteria in a healthy microbiome. These anticancer bacteria contribute to tumor control by inhibiting histone deacetylases and activating calcineurin [20].

Studies have demonstrated a significant reduction in proinflammatory cytokines, such as interleukins 6, 10, 12 and 22. This reduction is associated with a decreased risk of postoperative complications, thanks to strains of *Lactobacillus* and *Bifidobacterium*. Furthermore, analyses performed on mice indicate that FMT effectively reduces pathogenic species, structurally restores intestinal microbiota, prevents bacteremia and mitigates intestinal adverse effects from certain chemotherapy treatments, including diarrhea and mucositis [21].

The aim of this study is to summarize the key information from the literature that clarifies the impact of fecal microbiota transplantation in colorectal cancer, the mechanisms through which it acts and the expected outcomes from this particularly intriguing treatment.

## 2. Gut Microbiota and Colorectal Cancer

### 2.1. Composition of Gut Microbiota

The “Hidden organ”, as the microbiome is named, is made up of *Firmicutes* and *Bacteriodetes*, the dominant bacterial phyla, as well as Proteobacteria, Fusobacteria, Actinibacteria and Verrucomicrobia [22]. The microbiome’s role within the host includes interactions with the intestinal epithelial barrier or superficial cells, which can, in turn, expose the host to various extraintestinal effects. This can lead to cardiovascular conditions such as atherosclerosis, hypertension, heart failure, myocardial infarction and even stroke [23]. Table 1 presents the oncogenic potential of specific bacterial phyla that are representative of the gut microbiome in particular patient subtypes.

### 2.2. Role of Gut Microbiota in Colorectal Cancer Development

The external tissue surface plays a crucial role in the body’s immune response to the microbiome, largely due to the intestinal component’s sensitivity and responsiveness to external factors. The immune system plays a critical role in defending the body against microbes that enter from external sources. This process relies on “eager fighters”, or receptors, that develop resistance to the extrauterine environment and establish harmony with the intestinal microbiome [27]. In a pathophysiological context, the intestinal bacterial community can enter a state of dysbiosis, resulting in alterations to the gut composition and disrupting the host’s overall homeostasis [28]. The aggressive factor triggers an inflammatory response by increasing the number of neutrophils, which not only act at the site of inflammation but also affect the surrounding mucosal tissue by producing pro-inflammatory cytokines [29]. The gut microbiome also produces a large amount of ligands for pattern recognition receptors (PRRs), which have the role of protection against aggressors, maintaining abundant flora [30].

The altered microbiome in colorectal cancer patients directly impacts the metabolism of other pathological processes due to the presence of pro-inflammatory bacteria. For instance, the increased risk of colon polyps was shown to be associated with elevated *Bacteroides*, while bacteria that produce bile salts and hydrogen sulfide raise the risk of colorectal cancer. In patients with colorectal cancer, an increase in *Escherichia coli* has been observed, which, through the polyketide synthase (pks+) genomic island, influences DNA alkylation and promotes tumorigenesis. Meanwhile, other bacterial structures, such as *Lactobacillus* and *Eubacterium*, have protective functions [31]. One of the critical changes observed in colorectal cancer patients is the transition from cellular oxidative metabolism to rapid glycolysis, along with various genetic alterations. Consequently, butyrate, which serves as the primary bacterial-derived energy source for colonocytes, is not oxidized as it is in a healthy state, leading colon tumor cells to rely on glucose for energy [32]. From a metabolic point of view, short-chain fatty acids (SCFAs) reduce the risk of developing colorectal cancer by maintaining an intact intestinal function, their main representative in this regard being butyrate, obtained from dietary fiber by fermentation [33]. The mechanism by which butyrate fulfills this function involves the activation of AMP, a precursor that activates the protein kinase AMP-activated protein kinase (AMPK), which regulates cancer cell proliferation and energy metabolism [34].

### 2.3. Dysbiosis and Carcinogenic Microbes

During the process of tumorigenesis, the gut microbiome demonstrates its important role based on the idea that genetic changes, along with environmental factors, are largely responsible for the development of neoplasms. The dysbiosis of the microbiome is caused by the imbalance of these environmental, dietary and lifestyle factors, which often leads to the onset of cancer [35]. All in all, the confluence of environmental factors, the gut microbiome and the internal gut environment under conditions of dysbiosis is an explanation for the early onset of colorectal cancer (EOCRC). Intestinal dysbiosis is caused by inflammatory processes resulting from the infiltration of mediators and cytokines after intestinal damage by microbes such as *Escherichia coli*, *Bacteroides fragilis* and *Fusobacterium nucleatum* [36]. According to studies in the literature, samples from colorectal cancer patients may contain not only bacteria but also *Candida albicans*, *Ascomycota*/*Basidiomycota*, *Aspergillus rambellii*, *Phytophthora capsici* and *Erysiphe pulchra*, as well as *Human Papillomavirus* and *Cytomegalovirus* [37].

Wang and collaborators are exploring hypotheses regarding the specificity of certain microorganisms in the intestinal environment of colorectal cancer. They note that generalizations made by excluding the unique characteristics of each population can lead to unrealistic results. According to their cohort study, they demonstrated that Chinese patients differ from those in Central Europe, both in microbiome structure and in neoplastic progression. Chinese patients demonstrated a higher prevalence of intestinal *Fusobacteria*, notably featuring microbes such as *Actinomyces*, *Flavonifractor plantii* and *Schaalia cardiffensis*. They also observed an increase in *Fusobacterium nucleatum* in advanced intramucosal carcinoma and a proliferation of *Actinomyces odontolyticus* at various stages [38].

Amitay and his collaborators found, through a comparison of healthy individuals and colorectal cancer patients across a sample of 10 studies, that Fusobacterium is the most common and indicative type in those affected by cancer [39]. Meanwhile, Liu demonstrated using mice that the microbiome is essential for the development of colorectal cancer in the context of dietary iron overload, as excessive iron creates an oxidative imbalance that promotes tumorigenesis [40].

Moreover, recent studies not only demonstrate differences in microbiota composition based on the stage of colorectal cancer but also suggest that the composition of tumor and gut microbiota may serve as a biomarker for disease progression or cancer risk. Furthermore, they reveal significant distinctions between the microbiota of the right and left colon, with each showing unique characteristics that may influence cancer development in different ways. Thus, the right colon appears to be more prone to tumor development due to its local microbiota composition. However, once cancer develops, it tends to induce a nearly identical microbiota composition in both sections of the colon [41,42,43,44].

## 3. Fecal Microbiota Transplantation: Principles and Procedures

### 3.1. Definition and Short History of FMT

FMT is a strategy for treating diseases related to intestinal dysbiosis and also serves as a scientific tool for researching microbiota in living organisms [45]. Given that alterations in the gut microbiota are a fundamental pathophysiological factor in the onset and progression of gastrointestinal diseases, FMT, despite appearing primitive, remains the only treatment method capable of reintroducing a healthy microbial ecosystem into the gut [46].

Historically, fecal transplantation has evolved through different periods. It traces back to 4th century China, where the book “Zhou Hou Bei Ji Fang” highlighted the consumption of fermented feces as a treatment for mild intestinal imbalances. In the first half of the 20th century, enemas using fecal water were employed for therapeutic purposes, marking the introduction of FMT in the English literature. By 2012, the use of frozen microbiota to treat *Clostridioides difficile* infection started to be reported as an effective method in the United States. In 2015, research in China continued to evolve, modernizing and updating the transplantation process [47].

### 3.2. Donor Selection and Screening

**Figure 1:**
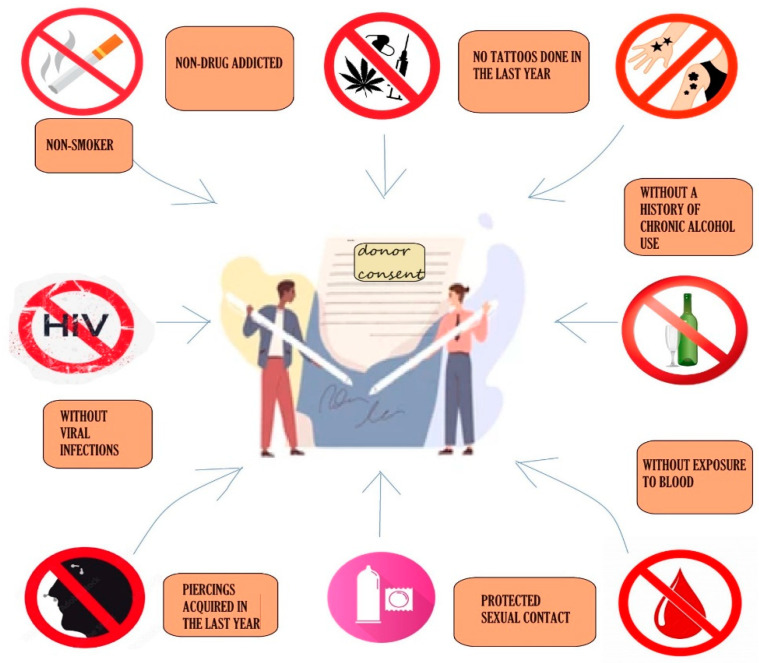
Some contraindications for the eligible donor in FMT.

The success of FMT is initially determined by the donor–recipient relationship, including their compatibility, adherence to treatment and willingness to accept this innovative method compared to other types of transplantation. An essential factor in this selection process is screening, as less than 20% of all potential donors are deemed suitable for the procedure [48]. Donor screening encompasses several conditions, beginning with a physical examination and a review of personal medical history, following a process similar to that for blood donors. Donors of legal age must provide informed consent, while minors require both their consent and that of their parents. Donors are not allowed to consume toxic substances, have tattoos, piercings, infectious symptoms in the last month, exposure to blood in the last year or a hereditary history of colorectal cancer and other gastrointestinal diseases. Potential donors have their blood drawn for virus testing (HIV, hepatitis, Epstein–Barr and others), antibodies and certain serologies, and then their stool is tested [49]. Figure 1 presents some of the main contraindications for donors.

### 3.3. Preparation and Administration of FMT

Stool sampling is the next step after screening and donor selection and is performed with a special kit without blood, urine or water along with the collected product. Currently, various methods are available for preparing biomaterial for FMT, though no standardized protocol exists. The literature describes several preparation techniques, which vary depending on the chosen method of administration and the preferences of the physician. For the donor, there are two options, the one to donate at home, only if the feces are kept at a low temperature and transferred within an hour after stool removal to the collection center, and the second option, donating directly to the center [50]. Although the volume of the microbiome is not directly proportional to the amount of stool, it is considered that 60 g of feces are sufficient for transplantation. Homogenization of the fresh stool is the next step, with 0.9% normal saline, in a ratio of approximately 1/2.5, obtaining a suspension. The undigested food must be removed from this suspension, which involves passing it consecutively through stainless steel sieves of different millimetric sizes [51]. A homogeneous liquid specimen will be obtained and utilized for transplantation in its fresh state within sterile syringes or alternatively, preserved in a frozen form. The sample may be administered using fecal capsules or through a refined washed microbiota preparation, facilitated by an automated purification system specifically designed for washed microbiota transplantation. Fresh samples should be utilized within six hours of collection or preserved at −80 °C until required. Prior to storage, samples should undergo additional filtration steps and be maintained in glycerol for optimal preservation [19]. The capsule form involves mixing the obtained suspension of feces with glycerol and about 20 min centrifugation at room temperature. When the supernatant is obtained, it must be centrifuged for about 30 min at a temperature of a few degrees Celsius, but also at a higher speed. In order to be individually encapsulated (template, gelatin or handmade, individually) the liquid obtained is separated from the supernatant which will be discarded and mixed with the residues. Finally, the capsules are frozen and kept for up to 60 days [52].

Another process involves fecal microbiota washing, where the suspension obtained from donor feces mixed with sterile saline is centrifuged for 3 min to separate it from the supernatant. The result will be washed 3 times with sterile saline solution and the suspension can be used fresh or frozen, the latter requiring high water temperature for thawing. Microbiota transplantation is performed for the upper intestine orally, using capsules or a nasogastric tube. For the middle intestine, endoscopy and nasojejunal probing or a transendoscopic enteral tube (TET) are utilized. The lower intestine is treated through a colonoscopy, colonic TET or enema [53].

### 3.4. Safety and Efficacy of FMT

Over a 20-year period beginning in 2000, approximately 1.4% of patients who underwent FMT experienced death or serious adverse reactions. However, these incidents predominantly occurred in patients with a pre-existing compromise of the mucosal barrier [54]. For example, in inflammatory bowel disease, according to meta-analyses and reviews, FMT is a relatively safe method, with adverse reactions not being reported with a significant increase. Often, the adverse effects of FMT consist of mild gastrointestinal reactions (such as meteorism, nausea, vomiting, diarrhea or constipation), but in principle, it is considered an effective and safe remedy [55].

## 4. FMT in the Management of Colorectal Cancer

### 4.1. Preclinical Studies

In order to demonstrate the effects of FMT on colorectal cancer, Yu and colleagues used a mouse model. Healthy mice were injected with sodium dextran sulfate and azomethane to induce colorectal cancer, after which they received FMT via enema from healthy mice. The results were promising, showing suppression of colorectal cancer progression due to the immune response. This included significant extravasation of CD49d+ NK and CD8+ CD49d+ NK cells, which have a direct effect on proliferative cells, as well as a reduction in immunosuppressive Foxp3+ Treg cells. Additionally, the regulation of interleukins 6, 12a and 12b, among others, suggests that managing microbial dysbiosis may be one of the most important effects [56]. From the perspective of Zhang and collaborators, tests in mice indicate that FMT offers benefits for enhancing the response to immunotherapy in advanced cancer patients. Specifically, an increase in CD8+ T-cells was observed following treatment, along with greater responsiveness to anti-PD-1 therapy and improved symptomatology in the mice [57]. Chen argues that mouse models often lack accurate similarities to human conditions because they are not implanted with human colon cancer cells. He suggests that a more reliable method could involve implanting human cells, which could be achieved either surgically or non-invasively through transanal injection into the submucosa [58].

### 4.2. Effects of FMT

The therapeutic effect of FMT, following the selection and preparation protocol via enema in approximately 400 patients with gastroenterological, neurological and cancer conditions, was positive and free of side effects. It is important to note that the transplantation solution was adjusted with NanoGAS water, which served to enhance the multiplication of bacterial colonies. Tanaka and co-workers applied this adaptation of FMT with NanoGAS water in the treatment of a male patient with stage IV pulmonary adenocarcinoma, administering it once a week for six months. As a result, the patient’s life was extended by an additional year following the transplant. More specifically, the noticeable changes were the strengthening of immunity, changes in the microbiome related to its variety, better tissue plasticity and more qualitative carbohydrate and lipid metabolisms [59]. Because of the limited information on the immune-targeted effect of the gut microbiome against colorectal tumors, Dingenen and his study partners are highlighting the anti-PD-1 efficacy in relation to the amplified proportion of *Lachnospiraceae*, *Lachnoclostridium* and *Flavonifractor* bacteria in gastrointestinal neoplasms, in a cohort study [60]. Clinical studies indicate that there are certain risks associated with fecal transplantation, particularly regarding the non-selective transmission of other pathologies, such as obesity or atherosclerosis. These findings underscore the need for a rigorous and thorough donor selection process. One notable case involved a woman who became obese following the transplant, mirroring the donor’s condition, as the host’s metabolism was transferred [61].

Numerous clinical studies have identified the microbiome as a potential tumor marker in colorectal cancer patients, those at risk of developing the disease and healthy individuals. By assessing the levels of *Fusobacterium nucleatum* or oral markers, it may be possible to enhance the prognosis for these patients [62]. Dai addresses the practical challenges of using FMT for colorectal cancer patients, including potential human errors during colonoscopy, anesthesia risks, time pressures for interpretation and the costs associated with CT scans. He also highlights the potential of FMT DNA testing as a colorectal cancer screening option to be explored for future application [63]. It is important to highlight that, while certain bacterial species and their variations are being explored for associations with tumor pathologies, the evidence remains insufficient to justify targeting gut microbiota in anticancer treatment.

### 4.3. Potential Mechanisms of Action

In recent years, studies have indicated that in colorectal cancer, the rate of neoplastic cell proliferation is driven by chronic inflammation. It has also been observed that an increase in microbial diversity can trigger the programmed cell death of neoplastic cells. However, the precise mechanisms underlying these effects remain unclear [64]. A balance of microbial types is considered necessary to limit inflammation, both prophylactically and curatively [65]. Transplanted gut microbes act on the immune system by controlling cancer antigens, as well as natural and acquired immunity [66]. The transplanted microbiome will act not only on tumorigenesis but also as an adjuvant to immunotherapy by mediating the response to the treatment. Weakening the host’s immune checkpoint inhibitor (ICI) resistance, altering the tumor-related ecosystem and controlling immune–tumor cell interference are the mechanisms by which FMT influences the response to immunotherapy [67].

The gut microbiome contributes to the metabolic changes produced by cancer and chronic inflammation by being involved in the biochemical processes of transforming nutrients and obtaining other biologically active products. By obtaining short-chain fatty acids (SCFAs), which are found almost entirely in the colon and which are produced by bacteria through the fermentation of starch and dietary fiber, the microbiome proves its role in metabolism. Butyrate, as a significant representative of SCFA in the large intestine, overtakes glucose as an energy source used by cells, but in cancer-altered phenomena, such as the “Warburg effect”, where physiological energy sources are replaced and aerobic glycolysis is used, it acts differently. The modified action of butyrate will be to regulate apoptosis and uncontrolled cell growth as well as enhance cancer protection by maintaining homeostasis [68].

FMT is increasingly regarded as a viable approach for restoring microbial balance and functionality in the context of various pathologies [69]. Because the health of the microbiome predisposes the host’s organism to maintain the exercise of the intestinal barrier function, FMT can be a solution to restore this balance by transferring healthy microbial flora to an organism in which this role has been disrupted due to pathogenesis [70].

## 5. Challenges and Considerations

The number of colorectal cancers has reached alarming levels globally, with concerning incidence rates [4,71,72,73,74]. Despite these concerning statistics, some patients consider FMT as an alternative medicine option, while others are willing to pursue it in order to avoid more invasive procedures, especially after multiple unsuccessful treatment attempts. In their search for an optimal solution, many patients rely on the recommendations of their attending physicians, while others proactively educate themselves and prepare for the procedure [75]. Al-Bakri examined the perceptions of Jordanian health specialists regarding FMT, noting concerns about potential threats to patient dignity. Approximately half of the study participants expressed religious reservations about the procedure [76]. Another study, no older than 5 years old, shows the attitude of new generations of Chinese doctors about this treatment. The participants, who were first-year resident physicians, largely rejected the method even in hypothetical situations where they had a disease that could benefit from FMT, despite their doctor’s recommendation. The primary reason for this rejection was a lack of information [77].

Baunwall et al. investigated the widespread use of microbiome transplantation across Europe. Their study confirmed that, among 31 centers, colonoscopy was the preferred delivery method for treating *Clostridioides difficile* infection. Additionally, more than 70% of these facilities employed a single treatment protocol with multiple administrations at predetermined time intervals. Furthermore, the majority of these centers used frozen material for FMT rather than fresh samples, with medical observation following the procedure lasting approximately two years [78]. The use of FMT was classified as a medicine under the Human Medicines Regulations 2012 in England, potentially influencing the opinions of health specialists [79]. The standardization of FMT could offer significant advantages in oncology by promoting microbial diversity and enhancing epithelial protection, as suggested by cohort studies involving patients with neoplastic conditions, despite the doubts arising from theoretical considerations [80].

### 5.1. Long-Term Safety and Monitoring

Studies in the literature highlight the need for the close monitoring of patients who have undergone FMT to evaluate their progress concerning potential theoretical risks. Recent findings on the well-established positive effects of FMT, particularly in treating recurrent *Clostridioides difficile* infection, confirm its safety in a growing number of patients worldwide. The short-term safety of FMT is well-documented, with a reasonable safety margin observed in immunosuppressed individuals, including those with cancer [81]. In a study by Zou and colleagues, conducted at a single center with around 25 pediatric patients from a total of just over 70, the long-term efficacy of FMT showed a decline over a five-year period. Nonetheless, the study confirmed that FMT is a safe therapeutic option for microbiome imbalances in children. It also indicated that quarterly repeat treatments may be needed for recurrent conditions [82].

Another study found that all nine adult responders to FMT treatment for recurrent active ulcerative colitis experienced positive long-term outcomes, with no infections or adverse effects, likely due to the absence of additional comorbidities [83]. Research on the relationship between the microbiome and colorectal cancer is ongoing, aiming to move beyond animal models and develop a broader future perspective through technological advancements. The goal is to personalize transplantation therapy, reduce ambiguity and enhance the understanding of both the immediate and long-term effects on this patient population [84].

### 5.2. Ethical and Regulatory Issues

The limited data on the long-term outcomes of FMT across various pathologies raise concerns about the susceptibility of the donor–recipient complex, especially in patients with underlying conditions. These concerns encompass emotional instability, potential health risks, fear of exploitation, rigid donor selection criteria and challenges related to obtaining informed consent [85].

Discussions about uncertainty are amplified by the internet, especially with the viral spread of do-it-yourself transplant practices accessible to the public. Chinese researchers have observed that skepticism often stems from gastroenterologists and internists rather than from the patients themselves.

Some patients express openness to FMT and show a willingness to undergo this treatment, hoping it will improve their disease outcomes [86].

However, the vulnerabilities of colorectal cancer patients must not be overlooked in these decisions. Factors such as pain, cancer-related fatigue, increased susceptibility to infection and impacts on quality of life, including sexual health, the interruption of professional activities and financial issues, are significant considerations [87]. The key concept is the recognition of the challenging battle that patients face against a relentless disease, which has a high mortality rate and causes significant suffering.

## 6. Discussions: Future Directions and Perspectives

### 6.1. Potential for Personalized Medicine

The genetic and non-genetic diversity of colorectal cancer contributes to reduced medication response and complicates treatment, especially when histopathological examinations show tumor formations with similar characteristics but differing molecular responses. This basis of genomic diversity reinforces the need for personalized therapy. Personalized medicine focuses on the genetic variations within colorectal cancer, incorporating the patient’s medical history to analyze and obtain detailed insights into the unique characteristics of their tumor. This approach aims to overcome the cancer patient’s resistance to conventional therapies [88,89] Another advantage that targeted therapy offers is the onset of treatment in the early stages of the disease, which obviously improves patients’ survival rates [90]. Integrating the microbiome into personalized therapies leverages its capacity to adapt and influence pathogenesis, phenotyping, host responses to treatment and disease progression, highlighting its potential in cancer treatment [91].

### 6.2. Integration with Other Therapies

In the treatment of colorectal cancer, the basic therapies have been surgery, radiotherapy and chemotherapy, but since the second millennium, with the inclusion of targeted therapies with bevacizumab and cetuximab, the curative horizon has widened for this type of cancer [92]. In order to integrate FMT as an adjuvant therapy in colorectal cancer, rigorous donor and patient selection, seriousness of screening, adjustment of microbiome transmission and anything else that might pose an additional risk of complications for immunosuppressed patients must be considered. An important consideration is the preference for autolog FMT, which may potentially be a means of colorectal cancer recurrence, although it promises a lower rate of disease transfer. An important aspect to analyze is the time required for harvesting and preparing the transplant material in relation to initiating cancer treatment and determining the most effective route of administration. When the intestinal component is vulnerable to damage, colonoscopy is not recommended. Similarly, using the upper intestine poses concerns about fragility and oral mobility. Therefore, the most effective options remain internal administration and enema [80].

The microbiome’s ability to predict the body’s response to immunotherapy is critical for understanding the relationship between microbiome health and immunotherapy outcomes. This predictive power stems from the unique composition of each individual’s microbiome, which is shaped by external factors and the environment. This individuality allows for the classification of patients into responders and non-responders to immunotherapy. Furthermore, the microbiome’s role in enhancing immunity, through the differentiation of natural killer cells, regulatory T cells (Tregs) and lymphocytes, further underscores its importance in determining immunotherapy effectiveness [93]. Resistance to immunotherapy and adverse reactions continue to pose significant challenges in the administration of treatments. The role of microbiota transfer remains uncertain. However, emerging evidence suggests it may facilitate a reduction in resistance to immune checkpoint inhibitors and lessen complications associated with therapy. The mechanism that facilitates the response to immune checkpoint inhibitors (ICIs) and enhances their treatment efficacy is based on the premise that alterations in the microbiota can induce changes in the tumor microenvironment. These changes may improve the immune system’s ability to recognize and target cancer cells more effectively [67,94].

### 6.3. Advances in Microbiome Research and FMT Technology

The human microbiome is a subject of great interest in modern medicine, especially for its prophylactic and curative role in various diseases, which is why it requires more significant data transparency and more careful study for development in current hospital practice [95]. The knowledge of the microbiome has been optimized in recent years by the evolution of sequential and multi-omics technology. Modern procedures aim at taxonomy and shotgun metagenomic sequencing and the metagenomic shotgun sequencing of the rRNA16s gene, so that microbiome compounds can be studied more closely and under more financially permissive conditions, but with promising results. All these actions must be supported by a qualitative database, with the expectation of limiting errors [96]. The abundance of microbiome research in animals has generated a substantial amount of knowledge about this ecosystem, but it has also come with disadvantages: a long study time, incongruence between human and animal cells and high costs. The current possibility to study the microbiome in a controlled, in vitro environment is due to the engineering of biomaterials and microfluidic tools, which have proven to be more useful than classical methods [97].

### 6.4. Controversies

The debates surrounding FMT primarily center on the method’s safety, patient outcomes and potential long-term adverse effects of the procedure. Specifically, concerns arise from the limited number of case reports and series that substantiate the safety of FMT in immunocompromised patients, where the potential transmission of *Epstein–Barr virus* poses significant management challenges, potentially avoidable with more stringent donor screening practices. Then, there is the recipient’s exposure to autoimmune diseases, overweight and other conditions that can be transmitted through the microbiome [98]. Controversy also arises over stool bacteria that are not only found more frequently in the feces of cancer patients compared to non-cancer patients but also favor disease progression, according to in vitro and in vivo animal studies, an example being the *Parvimonas micra* population [99].

### 6.5. Future Research Directions

Chen and Chiu view the replacement of oral administration of microbial compounds with colonoscopy as a promising future approach for FMT delivery, advocating for further studies on this treatment method [100]. In direct relation to the availability of study findings, Borody et al. suggest that the indications for FMT therapy should evolve to become more accessible [101]. No later than 2023, Wang highlighted the importance of further investigating FMT in capsule form, aiming to clarify the link between the microbiome and FMT outcomes, which may be affected by various factors [102]. To fully establish and standardize the potential benefits of FMT in tumor pathology, it is crucial to conduct prospective double-blind studies in accredited and controlled research centers. These studies are essential for providing robust evidence and ensuring the reliability of FMT’s therapeutic effects.

## 7. Conclusions

The potential of FMT as a therapy for colorectal cancer is significantly supported by literature, demonstrating its efficacy in gastrointestinal disorders such as *Clostridioides difficile* infection, ulcerative colitis and irritable bowel syndrome, among others. The potential of FMT for colorectal cancer is reinforced by the collaborative efforts of researchers exploring its mechanisms and benefits, despite a notable lack of human studies specifically focused on this cancer type. The significance of the gut microbiome in diagnosing colorectal cancer, along with its influence on the disease’s onset and progression, underscores the need for new findings aimed at individualizing and standardizing this treatment.

## 8. Material and Methods

Original reports and reviews describing FMT were reviewed using manual and electronic bibliographic sources. English articles were searched for in the Web of Science, PubMed, Medscape, Up-to-Date and Google Scholar databases up to 2024. In this study, 102 publications were selected. The list of alternatives used for searching databases includes “fecal”, “faecal”, “microbiota”, “cancer”, “colorectal”, “microflora”, “feces”, “faeces” and “stool”, individually or with the following options for the transplant lexical field: “transplant”, “donor”, “donation” and “enema”. In order to ensure a complete search result, all of these terms were searched for independently as well as in combination with various descriptive terms such as “Colorectal cancer”, “Rectal cancer”, etc.

## Figures and Tables

**Table 1:** Oncogenic potential of key bacterial phyla in the gut microbiome across specific patient subtypes.

Bacterial Phyla	Representative Subtypes	Pro-Oncogenic	Anti-Oncogenic	References
Firmicutes	*Lactobacillus*, *Bacillus*, *Enterococcus*, *Ruminococcus*		+	[20,24]
Bacteroides	*Bacteroidetes*	+		[20,24]
Proteobacteria	*Escherichia*, *Desulfovibrio*	+		[20,24]
Fusobacteria	*F. Varium*, *F. Nucleatum*	+		[20,25]
Actinobacteria	*Bifidobacterium*		+	[20,24]
Verrucomicrobia	*Akkermansia*		+	[24,26]
